# The Intergenerational Transmission of Early Childbearing: Examining Direct and Indirect Associations in a Swedish Birth Cohort

**DOI:** 10.3390/bs9050054

**Published:** 2019-05-16

**Authors:** Robin S. Högnäs, Alessandra Grotta

**Affiliations:** 1 Department of Public Health Sciences, Stockholm University, 106 91 Stockholm, Sweden; alessandra.grotta@su.se; 2 Department of Medical Epidemiology and Biostatistics, Karolinska Institutet, Box 281, 171 77 Stockholm, Sweden

**Keywords:** intergenerational transmission, early childbearing, socioeconomic status, education

## Abstract

*Background*. Research shows that early childbearing is associated negatively with educational attainment and socioeconomic status (SES). Children born to young versus older mothers often do less well in school, and many have early first births. Some studies suggest that mothers’ early childbearing operates through SES to influence the daughters’ early childbearing, and some argue that the association is strong net of SES. The current study tests these direct and indirect associations. *Methods*. We estimate the pathways through which mothers’ early childbearing influences daughters’ early childbearing in several steps. First, we examine bivariate associations between mothers’ early childbearing and SES, followed by bivariate associations between mothers’ SES outcomes and their daughters’ early childbearing. We then estimate the average marginal effects (AMEs) of mothers’ early children on daughters’, and a KHB decomposition to examine direct and indirect associations. *Results*. Findings suggest both direct and indirect associations. Nested models show that, net of a range of SES characteristics, mothers’ early childbearing increases the probability of daughters’ by approximately 8%; and KHB results suggest 37% mediation, with daughters’ school performance (12%) and household educational attainment (10%) contributing the highest shares. *Conclusion*. Mothers’ early childbearing and subsequent SES collectively influence the long-term wellbeing of children. Thus, early childbearing has consequences both within and across generations.

## 1. Introduction

Most recent estimates from The World Bank suggest that early childbearing in Western countries has decreased significantly over the past few decades. In Sweden, where rates have been historically lower than many other Western countries, still over 5 births per 1000 were to women ages 15–19 in 2016 [1]. Even though there has been much progress in preventing teenage and early births [2], a nontrivial segment of Swedish girls bear children early, and are vulnerable to the risk factors associated with early childbearing. There are many risk factors associated with early childbearing, and the most salient may be socioeconomic vulnerability, and having a young mother [3,4,5,6,7,8,9,10,11]. Research further shows that early childbearing can alter young women’s life course trajectories in terms of educational attainment and employment [12,13,14,15,16,17]. Some research even suggests that early childbearing increases the risk of mortality [18]. For these and other reasons, scholars and policy makers have long been concerned with the causes and consequences of—and life course trajectories following—early childbearing [1].

Time investments shift from oneself to one’s children following the onset of parenthood. The attention and material, social, and emotional care necessary for positive child development [19] may pose great challenges for young parents to continue investments in education, particularly given that many young parents are from lower socioeconomic circumstances, e.g., [6]. Consequently, young mothers tend to achieve less education, work less, and overall end up in lower socioeconomic positions, compared to their counterparts who bear children at older ages [12,13,14,15,16,17]. In turn, daughters who are born to, and reared by, young mothers, grow up in less educated and low-income contexts, struggle in school [13,14], and many go on to become young mothers themselves [3,6,7,8,9,10,11]. Thus, socioeconomic and other disadvantages that come with early parenthood is often reproduced over generations. 

The linked lives—or interlocking of parents’ and children’s lives—begins at the birth of a child, and continues throughout child development and across the stages of adulthood [19]. Young children are fully dependent upon parents for attention, caretaking, and basic material and social resources [20]. Parents influence their children’s first words, their ability to read, to communicate, and among many other things, parents impart to children their views and values about religion, politics, education, and family formation [21,22]. Social learning theory [23] posits two ways that behaviors are adapted, either from one’s individual and direct experience, or through observation of behaviors. Bandura argues, “most behaviors that people display are learned deliberately or inadvertently, through the influence of example” [23] (p. 5). The theory further posits that observed behaviors are stored and often modeled over time. 

We argue that social learning underlies the process through which daughters adapt the mores, norms and behaviors of their mothers. Inadvertently, social cues [23] associated with investments in education, long-term financial wellbeing, and the timing of motherhood are passed from mothers to daughters. Over time, daughters begin to model the behaviors of their mothers. Indeed, the trajectories of young mothers are often transmitted across generations, and studies show that children of young mothers themselves become young mothers [7,8,9,10,11]. There is, however, some disagreement as to whether the association is direct or indirect. For example, consistent with Bandura’s social learning theory [23], an early study from the late 1970s [6] suggests that mothers and daughters share an early age at first birth because mothers serve as role models for their daughters. Later studies found that even after controlling for socioeconomic factors and other behavioral characteristics, daughters are more likely to have an early first birth if their mothers did [7,9,11]. Still, some research shows that the association between mothers’ and daughters’ early childbearing is explained by socioeconomic circumstances [8,10], and thus early childbearing among mothers more likely operates through socioeconomic status (SES) to influence their daughters’ early childbearing. 

There is evidence of both direct and indirect associations between mothers’ and daughters’ early childbearing. Stanfors and Scott [11], for example, tested whether socioeconomic characteristics were intervening mechanisms in the intergenerational transmission of early childbearing. They found that, net of mothers’, fathers’, and daughters’ education and income, daughters were significantly more likely to have an early first birth (by approximately age 21) if their mothers were young mothers. While Stanfors and Scott primarily found a direct association between mothers’ and daughters’ early first births, along with other studies [7,8,9,10,11], they did not test formally the direct and indirect influences of mothers’ early childbearing and SES on daughters’ early childbearing. 

While socioeconomic characteristics are salient in terms of the causes and consequences of early childbearing, additional factors may be important. For example, young versus older mothers have a higher risk of single parenthood [17], and research suggests that family structure instability during childhood can subsequently result in offspring’s early childbearing [24]. Also, teen pregnancy and early motherhood may increase the risk of experiencing mental health problems [25,26]. Even so, Mollborn and Morningstar [26] found that young mothers experienced similar levels of distress before and after childbearing. In other words, they did not find evidence that early childbearing increased distress. As the authors note, young mothers often have socioeconomically disadvantaged backgrounds, and this could account for young mothers’ pre- and post-childbearing distress. Thus, it is difficult to determine whether early childbearing causes distress (or other mental health issues), or whether those who have mental health problems are at greater risk of early childbearing. 

### Current Study Aims and Research Questions

This study extends the literature in several ways. First, we replicate prior studies examining the intergenerational transmission of early childbearing. Most prior studies have emphasized U.S. populations, our study examines the intergenerational transmission in a Swedish birth cohort. Second, little prior research on the Swedish population [11] has addressed the timing of first births over two generations. Moreover, studies have underscored the significance of SES status, welfare receipt, and daughters’ education as predictors, potential mediators, and outcomes of early childbearing [2,3,4,5,6,7,8,9,10,11,12,13,14,15,16,17,18]; however, no study of which we are aware has tested formally direct and indirect influences of mothers’ early childbearing on daughters’ early childbearing. Thus, in three stages, the current study examines the pathways through which early childbearing is transmitted from mothers to daughters. 

First, to establish whether outcomes from prior research [7,12,13,14,15,16,17] are associated with early childbearing in our sample, we examine bivariate associations between mothers’ early childbearing and subsequent socioeconomic status, familial context, mental health, and daughters’ school performance. Second, to determine whether outcomes associated with mothers early childbearing influence daughters’ early childbearing, we examine whether socioeconomic status, familial context, mothers’ mental health, and daughters’ school performance predict daughters’ early childbearing. Third, we use nested models to examine the intergenerational transmission of early childbearing from mothers to daughters. Finally, the current study uses mediation analysis to disentangle direct and indirect effects of mothers’ early childbearing on daughters’ early childbearing, underscoring the importance of household socioeconomic status and daughters’ education in the transmission. 

Specifically, this study examines the following questions: 1) Is mothers’ early childbearing associated with her subsequent socioeconomic status, family structure, mental health, and daughters’ educational performance?; 2) Do daughters reared in socioeconomically disadvantaged contexts in Sweden have a higher probability of bearing children at age 20 and younger?; 2) What factors mediate the association between mothers’ and daughters’ early childbearing? 

## 2. Methods

Data from the Stockholm Birth Cohort Study (SBC) allowed us to examine the association between mothers’ and daughters’ early childbearing, while examining mediators. The SBC was compiled in 2004 and 2005 using probability matching of two longitudinal data sources. The original data source was the Stockholm Metropolitan Study (SMS), which began collecting data on all 10-year olds and their families who lived in the Stockholm area in 1963 (*N* = 15,117). The SMS continued through 1985, and its purpose was to study stratification and mobility processes prospectively. Through probability matching, subjects from the original SMS were linked with the Swedish Work and Mortality Database (WMD), which includes those living in Sweden in 1980 and/or 1990, forming the SBC. Ninety-six percent of SMS participants were identified, and data were obtained about cohort members’ lives through 2009, resulting in a sample size *N* = 14,294 (see [27] for more details). 

Baseline data from the original SMS include mothers’ age at the first birth, information on parental co-residence in 1963, presence of household members with upper secondary school in 1966, mothers’ employment status in 1966, family’s social class in 1966, family’s welfare receipt between 1953 and 1965, mothers’ psychological problems between 1953 and 1965. Moreover, information was collected for the family’s attitudes toward higher education, which was summarized in a categorical score ranging from 1 to 10, and daughters’ school marks in the 6^th^ grade expressed as a categorical score ranging from 1 to 7. Information on cohort members’ deliveries are available up to 2002. We define early childbearing as having the first child at an earlier age than 21, both in mothers’ and in daughters’ generations. 

### Analytic Approach

Our goal was to examine whether early childbearing is transmitted over two generations and the pathways through which daughters have a higher probability of having an early first birth if their mothers had an early first birth. First, we computed descriptive statistics for the full analytic sample, and stratified by mothers’ age at first birth (i.e., 20 or younger, older than 20). We compared the distribution of baseline variables using chi-square and t-test for dichotomous and continuous variables, respectively.

We draw on prior research [7,8,9,10,11] to estimate bivariate associations between mothers’ early childbearing and household education, socioeconomic status and daughters’ education. We then estimate whether education, socioeconomic status and daughters’ education are associated with daughters’ early childbearing. Next, we estimate four logistic regression models, starting with a baseline model examining the bivariate association between mothers and daughters early childbearing. Model 2 adds mothers’ characteristics, Model 3 adds household socioeconomic status, and Model 4 adds variables associated with daughters’ education. Average marginal effects (AMEs) with 95% confidence intervals (95% CIs) are reported and compared across models. We report AMEs instead of odds ratios (ORs) as the former, contrary to ORs, are comparable across nested models [28].

Finally, we used the KHB method to decompose the total effect of mothers’ early childbearing on daughters’ early childbearing into direct and indirect effects mediated by mothers’ characteristics, socioeconomic status, and daughters’ education. KHB holds constant the variance in, and the shape of, the error distribution between the reduced and full models (without and with mediators), and is not affected by attenuation bias in nonlinear probability models [29]. 

Item missing on all covariates was less than 20%. Therefore, to retain more of our analytic sample, we used multiple imputation techniques to replace missing values for covariates only. We did not impute missing values for mothers’ nor daughters’ age at first birth. After selecting out non-missing cases for women who gave birth before 2002, our final analytic sample consists of *N* = 4834 women.

## 3. Results

Descriptive statistics are shown in Table 1. Among the 4,834 daughters in our analytic sample, who gave birth before 2002, 757 (approximately 16%) had their first child at age 20 or younger (and the earliest birth at age 15). Twenty-four percent of their mothers worked, and 90% were living with the daughters’ father in 1963. Eighteen percent of families received welfare support at some time between 1953 and 1965, 40% of the families were classified as working class, and 74% of daughters were reared in households in which no adult had an upper secondary education. Only 3% of the mothers reported psychological problems by the time daughters were age 12. After stratifying by mothers’ age at first birth, we observed a higher percentage of early childbearing among daughters whose mothers’ age at first birth was younger than age 21. Moreover, we observed that families in which the mothers had an early first birth versus a later first birth (i.e., after age 20) had significantly lower household education, a higher percentage of welfare receipt and more likely to be working class. 

**Figure 1:**
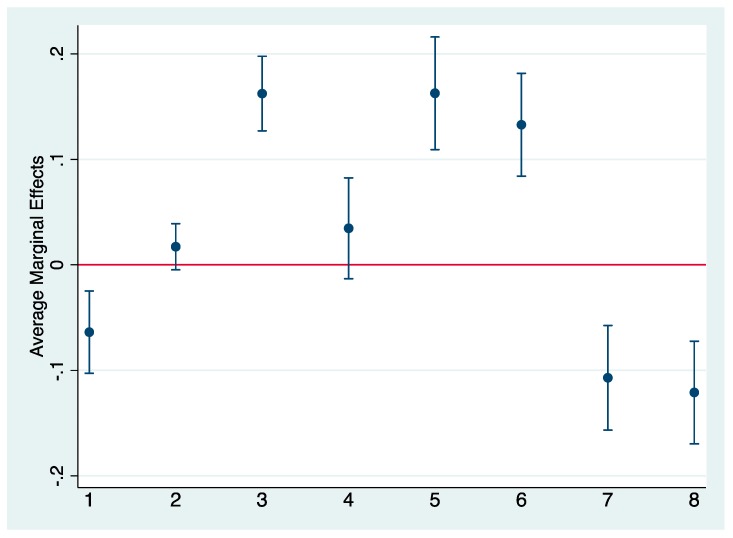
Bivariate Associations between Mothers’ Early Childbearing and Covariates. Note. All covariates are binary. 1 = Coresident Mother and Father 2 = Mother Had Psychological Problems; 3 = No Household Member with Upper Secondary School 4 = Mother Worked; 5 = Working Class; 6 = Family Received Welfare; 7 = Families Attitudes Toward Higher Education (range = 1–7); 8 = Daughters’ School Performance (range = 0–10).

**Figure 2:**
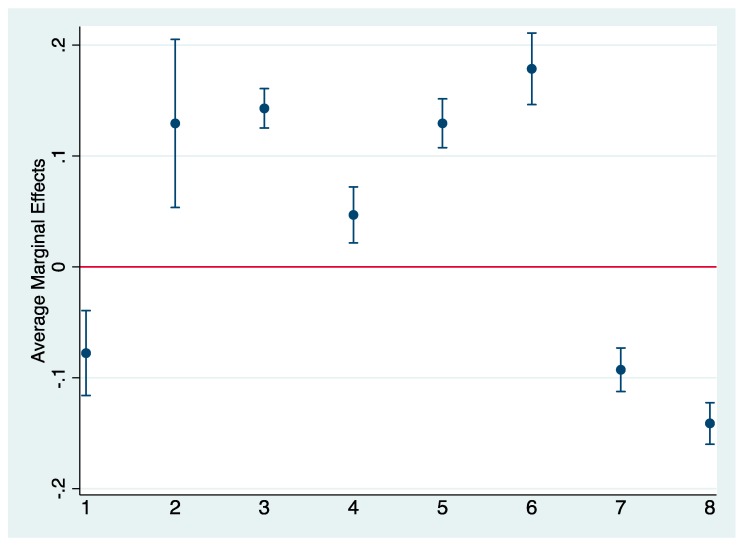
Bivariate Associations between Covariates and Daughters’ Early Childbearing. Note. All covariates. 1 = Coresident Mother and Father. 2 = Mother Had Psychological Problems; 3 = No Household Member with Upper Secondary School. 4 = Mother Worked; 5 = Working Class; 6 = Family Received Welfare; 7 = Families Attitudes Toward Higher Education (range = 1–7); 8 = Daughters’ School Performance (range = 0–10).

Next, because we are interested in the pathways through which mothers’ early childbearing influences daughters’ early childbearing, we estimate bivariate associations between mothers’ early childbearing and socioeconomic and other covariates (shown in Figure 1). We then estimate bivariate associations between socioeconomic and covariates and daughters’ early childbearing (shown in Figure 2). Results from our first set of bivariate analyses confirm significant associations between mothers’ early childbearing and co-residency with daughters’ father, mothers’ psychological problems, household educational level, working class, family’s receipt of welfare, family’s attitudes toward school and daughters’ school performance. We found no significant bivariate association between mothers’ early childbearing and mothers’ psychological problems nor mothers’ employment. AMEs shown in Figure 2, estimating the association between covariates and daughters’ early childbearing, suggest that all covariates were associated significantly with daughters’ early childbearing. 

Table 2 shows results from the nested logistic regression models. In Model 1, net of daughters’ birth order, there is a significant, 16% increase in the probability of daughters’ early childbearing (versus later childbearing) if their mothers’ age at first birth was age 20 or younger. Once mothers’ characteristics were added in Model 2, the AME decreased to 15%, suggesting that a very small proportion of the association between mothers’ and daughters’ early childbearing may be accounted for by mothers’ coresidency with fathers and mothers’ psychological problems. In Model 3, the probability that daughters bore their first child early if their mothers bore their first child early decreases from 15% to 9% with the addition of household socioeconomic status. Moreover, the AME decreases from 9% to 8% with the addition of characteristics of daughters’ education, i.e., the family’s overall attitudes toward higher education and daughters’ school performance, but remained statistically significant with a p-value less than 0.001 (in Model 4).

In our final analyses, Table 3 shows results from our KHB analyses decomposing the total, direct, and indirect associations between mothers’ early childbearing on daughters’ early childbearing. Results suggest that mothers’ early childbearing influences significantly daughters’ early childbearing (total effect OR = 2.41, *p* < 0.001), both directly (OR = 1.74, *p* < 0.001) and indirectly (OR = 1.38, *p* < 0.00). In terms of indirect associations, we estimate the percentage mediation of covariates that were significantly associated with mothers’ and daughters’ early childbearing (i.e., drawn from Figure 1 and Figure 2 and Table 2), i.e., household education, working class status, family welfare receipt, and daughters’ school performance. Results suggest that household socioeconomic status and daughters’ school performance together mediate more than 36% of the association between mothers’ and daughters’ early childbearing, with the highest percentage mediation accounted for by daughters’ school performance (approximately 12%) and household education (approximately 10%), followed by family welfare receipt (approximately 8%) and being working class (approximately 7%). 

## 4. Discussion

This paper examines whether early childbearing is transmitted from mothers to daughters, and the pathways through which daughters’ early childbearing is a function of mothers’ early childbearing. We extend prior research by using mediation analyses to test direct and indirect associations between mothers’ and daughters’ early childbearing in Sweden. We find that mothers’ early childbearing increases the probability of daughters’ early childbearing, net of the family structure in which daughters were reared, mothers’ mental health, household SES, and daughters’ educational characteristics. Specifically, net of these characteristics, daughters’ probability of early childbearing increased by about 8% if their mothers had an early first birth. We further found that household SES (i.e., low level of household education, working class, welfare receipt) and daughters’ school performance mediated part of the association (i.e., summing to more than 36% of the association). Our decomposition model suggests that the largest share of mediation was attributed to daughters’ school performance (12%), followed by low household education (10%), welfare receipt (8%), and working class status (7%). Overall, we found direct and indirect associations between two generations of young women’s (age 20 or younger) early childbearing. 

Our findings are consistent with, and extend, prior research [7,8,9,10], and we find more evidence of an intergenerational transmission of early childbearing in Sweden [11]. While the transmission of early childbearing from one generation to the next is mediated by household socioeconomic and school performance, there is also a direct association net of these characteristics [7,9,11]. Two simultaneous processes are likely at work here, selection and daughters modeling the behaviors of their mothers [15,22,23].

In terms of selection, mothers and daughters share socioeconomic disadvantages and this may contribute to both generations’ decisions about educational investments and the timing of childbearing. Wheeler [30] argues, for example, the conditions that often come with SES disadvantage (e.g., stress, living conditions) are barriers for low versus higher SES parents to invest in children’s education. These circumstances may be exacerbated when daughters rear their own children at young ages. Indeed, scholars have noted that the tremendous demands and lack of preparation for—and intensity of—becoming a parent are likely unforeseen to young women who are dependent themselves on adults [19,31]. Young mothers who do not have access to financial resources and/or strong supportive networks may attain less education than those who do. Moreover, along with prior research [17], our results suggest that daughters of young mothers are less likely to be reared by both parents, and recent research finds an associated wealth penalty over the life course [32]. 

In addition to the circumstances of low SES environments, from birth, daughters concurrently receive social cues [21,22,23] from their young mothers, and inadvertently, daughters model their mothers’ views, attitudes, and behaviors [23], including those related to educational attainment and having children. While we underscore findings that early childbearing is transmitted from mothers to daughters primarily through mothers’ SES and daughters’ subsequent school performance, it also may be transmitted directly. Parental attitudes are often adopted by children [22], and the timing of childbearing is no exception [33]. If young mothers have positive or neutral attitudes towards early childbearing, their daughters are subsequently more likely to adopt them [10]. Alternatively, daughters of mothers who delay childbearing for educational attainment may adopt less positive attitudes toward early childbearing from their mothers, and themselves delay childbearing to pursue higher education. 

As with all observational studies, this study is limited. First, our data did not allow us to account for socialization factors (apart from education) that may be relevant to early childbearing. For example, variation in parental attitudes towards contraception, sex education, and early family formation both within and between SES groups is likely important. In addition, we are not able to examine potential differences across cohorts. Changes in cultural attitudes toward women in higher education or increases in social mobility over time may influence the transmission of early childbearing from mothers to daughters. 

Despite these limitations, this study takes advantage of a prospective cohort study that includes information on a robust set of familial SES characteristics, family structure, and mothers’ mental health to assess the transmission of early childbearing. We were also able to use proxy measures for parental investments in daughters’ education with family attitudes toward higher education and school performance—both of which were measured prior to the youngest daughters’ (age 15) first birth in our sample. Moreover, these measures allow us to potentially capture important social cues passed from mothers (and fathers) to daughters [23], which may influence her future investments in education. In addition, the KHB decomposition allowed for a comparison with the results from our nested models, extending further the literature by testing the direct and indirect effects of mothers’ early childbearing on daughters’ early childbearing.

Future studies, where data allow, should prioritize contextual factors connecting two generations of early childbearing. There may be non-parental adult socialization processes that influence early childbearing from one generation to the next. Grandmothers or aunts, for example, may play a central role in the lives of young daughters, and variation in attitudes associated with family formation, sexual debut, and among others, the timing of childbearing may influence young women’s behaviors. This may be especially true if extended family assume childcare responsibilities or coreside with daughters. Future research may also explore the role of fathers in daughters’ and sons’ early childbearing in Sweden, and the mechanisms through which these intergenerational processes occur. It is also important for future research to examine potential cohort differences in the role of socioeconomic status in the transmission of early childbearing from one generation to the next, particularly given greater access to higher education (e.g., [11]). Lastly, systematic reviews or meta analyses using cross national (including Sweden) samples of studies may help to better understand the consistency of findings across cultural and policy contexts.

This study contributes to the extant literature by replicating previous studies [3,6,7,8,9,10,11] on the Swedish population [11], and more importantly by using mediation analysis to test formally the direct and indirect associations between mothers’ and daughters’ early childbearing in a context where few studies have examined the transmission (i.e., Sweden). In many ways, Sweden is set apart from many Western industrialized countries due to its policies aimed at gender equity and in support of parents. Thus, it is important to understand in this context how parents’ age at first birth and subsequent educational attainment and accumulation of financial and social resources collectively influence theirs and their children’s long-term wellbeing. While disadvantage tends to precede early childbearing, it may be possible that these disadvantages are exacerbated following early first births in Sweden. 

## Figures and Tables

**Table 1:** Descriptive Statistics for the Full Sample and Shown Separately by Mothers’ Timing of First Birth.

	Full Sample	Mothers’ First Birth ≤ Age 20	Mothers’ First Birth > Age 20	Sig ^a^
**Daughters’ Age of First Birth (%)**				
Age 20 or Younger	16	27	15	
Older than Age 20	84	73	85	***
**Daughters’ Birth Order (%)**				
1st Born	40	75	38	
2nd Born	35	21	36	
3rd Born	16	2	18	
4th Born or Higher	8	2	8	***
**Mother Worked (%)**				
Yes	24	27	24	
No	76	73	76	ns
**Coresident Mother & Father (%)**				
Yes	90	84	90	
No	10	16	10	***
**Family Received Welfare (%)**				
Yes	18	30	17	
No	82	70	83	***
**Mother Psychological Problem (%)**				
Yes	3	5	3	
No	97	95	97	ns
**Socioeconomic Status (%)**				
Working Class	40	55	33	
Middle Class or Higher	60	45	62	***
**Household Education (%)**				
No Household Members with Upper Secondary Education	74	89	72	
One or More Household Members with Upper Secondary Education	26	11	28	***
**Daughters’ Educational Performance (mean)**				
School Marks, 6th Grade (Range = 1–7) (sd)	5.11 (1.37)	4.80 (1.28)	5.14 (1.38)	***
**Family Attitudes Toward Higher Education** (mean) (Range = 0–10) (sd)	5.74 (2.39)	5.04 (2.42)	5.80 (2.37)	***
*N*	4834	359	4475	

* *p* < 0.05; ** *p* < 0.01; *** *p* < 0.001; ^a^ Chi-square tests where percentages reported; t-tests where means are reported. Note. Standard deviations are in parentheses for social marks and family attitudes toward higher education.

**Table 2:** Results from Logistic Models: Average Marginal Effects (AME) of Daughters’ Early Childbearing by Mothers’ Early Childbearing (*N* = 4834) ^1^.

	Model 1 AME (95% CI)			Model 2 AME (95% CI)			Model 3 AME (95% CI)			Model 4 AME (95% CI)		
Mothers’ Age of First Birth Age 20 or Younger	0.16	***	(0.11–0.21)	0.15	***	(0.10–0.20)	0.09	***	(0.05–0.14)	0.08	***	(0.03–0.12)
Daughter’s Birth Order												
2nd Born	0.04	**	(0.01–0.06)	0.04	**	(0.01–0.06)	0.03	*	(0.01–0.05)	0.02		(−0.01–0.04)
3rd Born	0.07	***	(0.04–0.10)	0.06	***	(0.03–0.10)	0.04	**	(0.01–0.08)	0.02		(−0.01–0.05)
4th or Higher	0.13	***	(0.08–0.18)	0.12	***	(0.08–0.17)	0.08	***	(0.04–0.12)	0.05	**	(0.02–0.09)
Coresident Mother and Father				−0.05	**	(−0.08–−0.02)	−0.01		(−0.04–0.02)	−0.01		(−0.04–0.02)
Mother Had a Psychological Problem				0.07	**	(0.02–0.12)	−0.00		(−0.05–0.05)	−0.01		(−0.06–0.04)
Household Socioeconomic Status												
No Household Members with Upper Secondary School							0.12	***	(0.08–0.15)	0.09	***	(0.05–0.12)
Mother Worked							0.02		(−0.00–0.04)	0.01		(−0.01–0.04)
Working Class							0.06	***	(0.04–0.08)	0.05	***	(0.03–0.07)
Family Received Welfare							0.08	***	(0.06–0.11)	0.07	***	(0.04–0.09)
Daughters’ Education												
Family’s Attitudes Toward Higher Education (Range = 0–10)										−0.00		(−0.01–0.00)
Daughters’ School Marks in 6^th^ Grade (Range = 1–7)										−0.04	***	(−0.05–−0.03)

* *p* < 0.05 ** *p* < 0.01 *** *p* < 0.001. ^1^ Confidence intervals (CI) are in parentheses. Reference categories are: for mothers’ and daughters’ age at first birth is older than age 20; birth order, 1^st^ born (which includes those with no siblings); mother and father did not live together; mother had no psychological problems; one or more household members with upper secondary school; mother did not work; middle class or higher; family did not receive welfare.

**Table 3:** Logit Models Decomposing (with KHB) Total Effects of Mothers’ Early Childbearing on Daughters’ Early Childbearing via Socioeconomic Characteristics and Daughters’ School Performance (*N* = 4834).

(ref. = Mothers’ First Birth Age 20 or Older)			
Mother Had First Birth Age 20 or Younger	Odds Ratio	Z Statistic	Sig.
Total Effect	2.41	6.32	***
Direct Effect	1.74	3.99	***
Indirect Effect	1.38	7.44	***
Percentage Mediation			36.66%
No Household Member with Upper Secondary School			9.98%
Working Class			6.70%
Family Received Welfare			7.61%
Daughters’ School Marks in 6th Grade			12.38%

* *p* < 0.05; ** *p* < 0.01; *** *p* < 0.001; Note. KHB model controls for all other variables.

## References

[1] The World Bank (2016). Adolescent Fertility Rate (Births per 1000 Women Ages 15–19). United Nations Population Division, World Population Prospects CC BY-4.0. https://data.worldbank.org/indicator/SP.ADO.TFRT?end=2016&locations=SE&start=1960&view=chart.

[2] Coyne C.A., D’Onofrio B.M. (2012). Some (but not much) progress toward understanding teenage childbearing: A review of research from the past decade. Adv. Child Dev. Behav..

[3] Rindfuss R.R., St. John C. (1983). Social determinants of age at first birth. J. Marriage Fam..

[4] Hongling X., Cairns B.D., Cairns R.B. (2001). Predicting teen motherhood and teen fatherhood: Individual characteristics and peer affiliations. Soc. Dev..

[5] Mersky J.P., Reynolds A.J. (2007). Predictors of early childbearing: Evidence from the Chicago longitudinal study. Child Youth Serv. Rev..

[6] Presser H.B., Miller W.B., Newman L.F. (1978). Social factors affecting the timing of the first child. The First Child and Family Formation.

[7] Card J.J. (1981). Long-term consequences for children of teenage parents. Demography.

[8] Kahn J.R., Anderson K.E. (1992). Intergenerational Patterns of Teenage Fertility. Demography.

[9] Manlove J. (1997). Early motherhood in an international perspective: The experiences of a British cohort. J. Marriage Fam..

[10] Barber J. (2001). The intergenerational transmission of age at first birth among married and unmarried men and women. Soc. Sci. Res..

[11] Stanfors M., Scott K. (2013). Intergenerational transmission of young motherhood. Evidence from Sweden, 1986–2009. Hist. Fam..

[12] Hofferth S.L., Moore K.A. (1979). Early childbearing and later economic well-being. Am. Sociol. Rev..

[13] Upchurch D.M., McCarthy J. (1990). The timing of a first birth and high school completion. Am. Sociol. Rev..

[14] Hofferth S.L., Reid L., Mott F.L. (2001). The effects of early childbearing on school over time. Fam. Plan Persp..

[15] Geronimus A.T., Korenman S. (1992). The socioeconomic consequences of teen childbearing reconsidered. Quart. J. Econ..

[16] Hoffman S.D., Foster E.M., Furstenberg F.F. (1993). Reevaluating the costs of teenage childbearing. Demography.

[17] Olausson P.O., Haglund B., Weitoft G.R., Cnattingius S. (2001). Teenage childbearing and long-term socioeconomic consequences: A case study in Sweden. Fam. Plan Perspect..

[18] Barclay K., Keenan K., Grundy E., Kolk M., Myrskylä M. (2016). Reproductive history and post-reproductive mortality: A sibling comparison analysis using Swedish Register data. Soc. Sci. Med..

[19] Elder G.H. (1998). The life course as developmental theory. Child Dev..

[20] Bowlby J. (1969). Attachment and Loss, Vol. 1 Attachment.

[21] Bengtson V.L. (1975). Generation and family effects in value socialization. Am. Sociol. Rev..

[22] Glass J., Bengtson V.L., Dunham C.C. (1986). Attitude similarity in three-generation families: Socialization, status inheritance, or reciprocal influence. Am. Sociol. Rev..

[23] Bandura A. (1971). Social Learning Theory.

[24] Hofferth S.L., Goldscheider F. (2010). Family structure and the transition to early parenthood. Demography.

[25] Maskey S. (1991). Teenage pregnancy: Doubts, uncertainties and psychiatric disturbance. J. R. Soc. Med..

[26] Mollborn S., Morningstar E. (2009). Investigating the relationship between teenage childbearing and psychological distress using longitudinal evidence. J. Health Soc. Behav..

[27] Stenberg S.-Å., Vågerö D., Österman E.A., Von Otter C., Janson C.-G. (2007). Stockholm Birth Cohort Study 1953–2003: A New Tool for Life-Course Studies. Scand. J. Public Health.

[28] Mood C. (2010). Logistic regression: Why we cannot do what do what we think we can do, and what we can do about it. Eur. Sociol. Rev..

[29] Breen R., Karlson K.B., Holm A. (2013). Total, direct, indirect effects in logit and probit models. Soc. Meth. Res..

[30] Wheeler S. (2018). The (re)production of (dis)advantage: Class-based variations in parental aspirations, strategies and practices in relation to children’s primary education. Educ. 3-13 Int. J. Prim. Elem. Early Years Educ..

[31] Hofferth S.L., Hofferth S.L., Hayes C.D. (1987). Social and economic consequences of teenage childbearing. Risking the Future: Adolescent Sexuality, Pregnancy, and Childbearing, Volume II: Working Papers and Statistical Appendices.

[32] Bernardi F., Boertien D. (2019). Childhood family structure and the accumulation of wealth across the life course. J. Marriage Fam..

[33] Keijer M.G., Liefbroer A.C., Nagel I. (2018). Adolescents’ expectations about the timing of family life events: Unraveling the role of value transmission and modeling. J. Fam. Issues.

